# Post-COVID-19 patients suffer from chemosensory, trigeminal, and salivary dysfunctions

**DOI:** 10.1038/s41598-024-53919-y

**Published:** 2024-02-11

**Authors:** Åsmund Rogn, Janicke Liaaen Jensen, Per Ole Iversen, Preet Bano Singh

**Affiliations:** 1 Department of Cariology and Gerodontology, Faculty of Dentistry, University of Oslo, Geitmyrsveien 71, 0455 Oslo, Norway; 2 Department of Oral Surgery and Oral Medicine, Faculty of Dentistry, University of Oslo, Oslo, Norway; 3 Department of Nutrition, Faculty of Medicine, University of Oslo, Oslo, Norway; 4 Department of Haematology, Oslo University Hospital, Oslo, Norway

**Keywords:** Post-COVID-19, Long COVID, Parosmia, Burning mouth, Dry mouth, Taste, Smell, Diseases, Oral diseases

## Abstract

Recent literature indicates that post-COVID-19 patients suffer from a plethora of complications, including chemosensory dysfunction. However, little attention has been given to understand the interactions between chemosensory, trigeminal, and salivary dysfunctions in these patients. The aims of this study were (1) to investigate the prevalence and combinations of chemosensory, trigeminal, and salivary dysfunctions, (2) to identify the odorants/tastants that are compromised, and (3) to explore possible associations between the four dysfunctions in post-COVID-19 patients. One hundred post-COVID-19 patients and 76 healthy controls (pre-COVID-19) were included in this cross-sectional, case-controlled study. Participants' smell, taste, trigeminal, and salivary functions were assessed. The patients had a significantly higher prevalence of parosmia (80.0%), hyposmia (42.0%), anosmia (53.0%), dysgeusia (34.0%), complete ageusia (3.0%), specific ageusia (27.0%), dysesthesia (11.0%) and dry mouth (18.0%) compared to controls (0.0% for all parameters, except 27.6% for hyposmia). Complete loss of bitter taste was the most prevalent specific ageusia (66.7%) and coffee was the most common distorted smell (56.4%). Seven different combinations of dysfunction were observed in the patients, the most common being a combination of olfactory and gustatory dysfunction (48.0%). These findings indicate that post-COVID-19 patients experience a range of chemosensory, trigeminal, and salivary disturbances, occurring in various combinations.

## Introduction

The COVID-19 pandemic is one of the most dramatic global health crises in recent history, presenting unparalleled challenges for societies all over the world. This infectious disease, caused by the novel coronavirus SARS-CoV-2, has spread rapidly affecting millions of individuals and causing substantial morbidity and mortality^1,2^. As of August 2023, there have been almost 1.5 million confirmed cases of COVID-19 in Norway and over 768 million worldwide^1^. COVID-19 seems to manifest in mild to severe forms, while some individuals remain asymptomatic^3^. The most common symptoms include fever, dry cough, fatigue, shortness of breath, sore throat, headache, myalgia, chills^4^ and loss of smell and taste^5–7^. Sudden loss of smell and taste is known to be the most discriminative symptom of COVID-19 infection^5–8^.

Most COVID-19 patients experience spontaneous recovery within weeks^9^ while others have persisting symptoms^10^. Patients with long lasting symptoms after a COVID-19 infection are often called post-COVID-19 patients. They are characterized by a history of probable or confirmed SARS CoV-2 infection, where symptoms persist more than three months after COVID-19 infection, with a duration of at least 2 months, that cannot be explained by another diagnoses. Moreover, symptoms may fluctuate or relapse over time and may persist from the initial illness, or as new onset following initial recovery^11^. Other names for post-COVID-19 are long-COVID and long-haul COVID. These patients present a variety of symptoms including smell and taste dysfunction^12^.

Angiotensin-converting enzyme 2 (ACE2) is identified as an entry protein for the SARS-CoV-2 virus into human cells^13^, and is expressed in the olfactory mucosa^14^, gustatory cells^15^, oral epithelial cells^16^, and salivary gland cells^17^. Reduced expression of ACE2 due to SARS-CoV-2 infection^18^ could be part of the explanation for the disturbances in smell, taste^15^, chemesthesis and salivary secretory rate.

Olfaction, gustation and chemesthesis are three independent modalities that are all important for the perception of flavor^19,20^. The sense of smell is mediated through the olfactory receptor cells located in the nasal epithelium that are innervated by the olfactory nerve^21^. The sense of taste is mediated through the gustatory receptor cells found in the taste buds in the oral cavity^22–25^. Taste buds are innervated by three cranial nerves: the facial, glossopharyngeal, and vagus nerves^22,26^. The sensation provided by the sensory fibers of the trigeminal nerve is called chemesthesis, which can detect temperature, touch, pain, and irritation. The multimodal interaction between the sensations of smell, taste and chemesthesis contribute to the overall flavor perception^19,20^. Furthermore, saliva plays an important role in dissolving solid foods and facilitating the binding of taste stimuli to adequate taste receptor cells^27^.

Taste loss can be difficult to differentiate from smell loss based on self-reporting alone due to the sense of smell being a large part of the flavor perception^7^. It is, therefore, important to supplement self-reporting assessment with objective testing. Chemosensory dysfunctions can be reliably divided into two main categories: quantitative and qualitative. The quantitative dysfunctions of smell and taste describe either complete loss (anosmia^28^, ageusia^29^) or reduced perception (hyposmia^28^, hypogeusia^29^), respectively. Specific ageusia is a rare condition where patients are unable to taste a specific taste quality. While diagnosing taste disorders, it is essential to distinguish between a total loss of taste (ageusia) or an attenuation of a specific taste quality (specific ageusia). On the other hand, qualitative dysfunctions reflect distortion of smell (parosmia, phantosmia) ^28^ and taste (dysgeusia) ^29^. Trigeminal dysfunction is broadly categorized into abnormal sensation without any pain (paresthesia) ^30^, or a painful, abnormal sensation with or without stimuli (dysesthesia) ^31,32^. Dry mouth includes a subjective feeling of dry mouth (xerostomia) and a condition of pathologically reduced saliva secretion (hyposalivation) as determined by sialometry^33^.

The most explored chemosensory dysfunction is parosmia, which has been found to be highly prevalent in post-COVID-19 patients^34–36^. Other conditions such as loss of smell (hyposmia^37^, anosmia^37^), loss of taste (hypogeusia^37^, ageusia^38^, specific ageusia^39,40^), other single taste disturbances^41–43^, altered taste (dysgeusia^44^), xerostomia^45^ and trigeminal dysfunction^37,46^ have also been reported in different sets of patients recovering from COVID-19. However, to our best knowledge, only one study has explored these dysfunctions in the same group of post-COVID-19 patients^45^. It is therefore of great interest to examine the occurrence of smell, taste, trigeminal and salivary dysfunctions in one common cohort, as these multimodal interactions may play a vital role in the intake of food in these patients. Altered smell, and taste sensation may lead to compromised nutritional intake, reduced quality of life and psychological distress^36^. Problems with reduced salivation^27^ and oral burning sensation^37^ may add further to these negative impacts.

The aims of the present study were therefore to (1) investigate the prevalence and combination of chemosensory, trigeminal, and salivary dysfunction in post-COVID-19 patients, (2) identify the tastants and odorants that are impaired due to COVID-19 infection, and (3) explore possible associations between the chemosensory, trigeminal, and salivary dysfunctions.

## Methods

### Study design and participants

This cross-sectional, case-controlled study was conducted at the Institute of Clinical Dentistry (ICD), Faculty of Dentistry, University of Oslo (UiO), Norway, between October 2020 and June 2023. Patients from all parts of Norway are referred to the university clinic for treatment of persisting smell, taste and trigeminal dysfunction. The “Clinic of Smell, Taste and Oral Pain” has recently been established at the ICD, dedicated specifically to help post-COVID-19 patients. Information about our services was provided through Facebook, newspaper articles, radio programs, national news channel on television and clinical workshop and seminar for ENT-surgeons, neurologists, dentists, and family doctors. One hundred post-COVID-19 patients referred to ICD were consecutively recruited for participation in this study. Seventy-six pre-COVID-19, healthy controls were used to ascertain that controls were not asymptomatic COVID-19 patients. These pre-pandemic controls had undergone the same assessments as the post-COVID-19 patients and were enrolled in previous studies at the ICD^47,48^. The exclusion criteria for controls were oral dryness, and presence of chronic diseases or medications that could affect smell, taste, trigeminal and salivary functions. The study was approved by the Norwegian Regional Committee for Medical and Health Research Ethics (REK 2021/274615) and was performed in compliance with the tenets of the Declaration of Helsinki. Written informed consent was obtained from all participants. The participants were instructed to refrain from eating, drinking, and smoking one hour prior to examination. The assessments of olfaction, gustation, trigeminal, and salivary function were carried out according to the protocol described below.

### The Oslo COVID-19 questionnaire

Participants’ medical history was obtained through standard health forms used at the university clinic at ICD. Post-COVID-19 patients then completed a questionnaire designed specifically for this study, called the Oslo COVID-19 questionnaire (Supplement). This questionnaire contained both binary, multiple choice and open-ended questions. In section 1 of the questionnaire, patients’ age, gender, occupational status, and use of tobacco was recorded. In section 2, information about COVID-19 infection was obtained; date of diagnosis, mode of confirmation of diagnosis (PCR test, home test, antibody test or clinical symptoms), and course of illness (mild, moderate, or severe). Finally, time for onset of loss of smell and taste, burning sensation and oral dryness was recorded (number of days before or after the confirmation of COVID-19 infection). Possible aetiology of chemosensory, trigeminal and salivary dysfunctions was also recorded (other viral or bacterial infections, menopause, trauma in head and neck region, head and neck surgery, dental surgery). In section 3, more specific questions about parosmia, dysgeusia, dysesthesia, and oral dryness were recorded as described below. This questionnaire did not assess phantosmia or phantogeusia.

### Olfactory assessment

Prior to objective olfactory testing, the participants were asked to score their smell perception on a linear visual analogue scale (VAS) from 0 to 10, where 0 indicated no smell perception and 10 very good smell perception. Score 5 was chosen as the cut-off point and scores 0– < 5 indicated low, 5– < 9 moderate, and 9–10 very good perception of smell, respectively. An Olfactory identification test was then performed using Sniffin’ Sticks test (Burghart Messtechnik GmbH, Holm, Germany). Twelve felt-tip odor pens were used for non-lateralized psychophysical testing of the olfactory function. The participants were informed about the procedure before the test started. The responses were recorded as either 1 = correct or 0 = incorrect and summated (score range 0–12). A normative classification^49^ was used to categorize participants into anosmic (score 0–6), hyposmic (score 7–10) and normosmic (score 11–12).

### Gustatory assessment

Prior to objective gustatory testing, the participants were asked to score their taste perception on a VAS from 0 to 10, where 0 indicated no taste perception and 10 very good taste perception. Score 5 was chosen as the cut-off point where scores 0– < 5 indicated low, 5– < 9 moderate, and 9–10 very good perception of taste, respectively.

Gustatory function was then measured using Taste Strips impregnated with solutions in four different concentrations of four different taste qualities: sweet, sour, salty and bitter (Burghart Messtechnik GmbH, Holm, Germany). The responses were recorded as either 1 = correct, or 0 = incorrect, and summated (score range 0–16). Participants were classified into ageusic (score 0), hypogeusic (score 1–8) and normogeusic (score 9–16) using a normative classification^50^. Participants were considered to have a specific ageusia if they were unable to detect all four different concentrations of one specific tastant.

### Assessment of self-reported parosmia, dysgeusia, dysesthesia and xerostomia

The categorization of parosmia was based on results from the questionnaire, where participants had either no parosmia (score 0), or parosmia (score 1). Further, if they had parosmia they were asked to describe how often they experienced parosmia: constantly, daily, sometimes, during meals, in between meals, or only when in contact with certain odorants. They were also asked to report which smells that were distorted from a list of 35 odors, as well as describe the character of the distortion.

Similarly, absence or presence of dysgeusia was categorized as follows: no dysgeusia (score 0), or dysgeusia (score 1). Participants with dysgeusia were asked how often they experienced dysgeusia (constantly, daily, sometimes, periodically, during meals, in between meals), and to describe the character of dysgeusia (metallic, rotten, harsh, salty, bitter, other).

Dysesthesia was categorized as either no dysesthesia (score 0), or dysesthesia (score 1). Participants reported how often they experienced dysesthesia (constantly, daily, sometimes, periodically, during meals, in between meals), asked to describe where in the mouth they experienced dysesthesia (whole tongue, anterior tongue, lips, palate, buccal mucosa, other), and identify food items that enhanced the dysesthesia (spicy, sweet, sour, salty, bitter).

Xerostomia was categorized according to participants' self-reported perception as either no dry mouth (score 0), or dry mouth (score 1). Participants were asked whether the symptoms of xerostomia started before or after COVID-19 infection and were asked open-ended questions where they could describe their experience of oral dryness. Finally, they were asked to report whether there were food items that they had to refrain from eating because of parosmia, dysgeusia, dysesthesia or xerostomia.

### Statistical analyses

The statistical analyses were performed using SPSS (SPSS Statistics version 28.0, IBM, Armonk, NY, USA), and Excel (Microsoft Excel version 2302, Microsoft, Redmond, Washington, USA). The results of descriptive analyses were presented as percentages, histograms and median/interquartile range (IQR)/range. Chi-square (χ^2^) or Fisher’s exact test were used to compare categorical variables and determine dependence between variables. A non-parametric test, Mann–Whitney U, was used to describe median differences between the groups in case of non-normal distribution. Pearson’s correlation coefficient (r) was used to measure the strength and direction of linear relationships between pairs. Statistical significance was considered at *p* < 0.05.

## Results

### Participant characteristics

The characteristics of post-COVID-19 patients and controls are presented in Table 1. No significant differences between patients and controls were found regarding age, gender, and tobacco use. Post-COVID-19 patients had a significantly higher number of other chronic conditions and used significantly more medications than controls. Most of the patients had experienced a mild or moderate course of COVID-19 infection, and only one of the patients reported a severe course involving hospitalization.

**Table 1:** Participant characteristics.

	Post-COVID-19 patients (n = 100)	Controls (n = 76)	p-values
Age (years)			
Mean ± SD	41.7 ± 12.9	41.8 ± 17.0	NS
Range	18.0–73.0	18.0–79.0	
Gender % (n)			
Female	68.0 (68)	73.7 (56)	NS
Male	32.0 (32)	26.3 (20)	
Chronic conditions % (n)			
Yes	40.0 (40)	10.5 (8)	˂ 0.001
No	60.0 (60)	89.5 (68)	
Allergy	24.0 (24)	18.4 (14)	
Heart disease	9.0 (9)	2.6 (2)	
Endocrinological disorders	8.0 (8)	0.0 (0)	
Auto-immune disorders	12.0 (12)	2.6 (2)	
Neurological disorders	5.0 (5)	2.6 (2)	
Psychiatric disorders	5.0 (5)	0.0 (0)	
Renal disease	1.0 (1)	0.0 (0)	
Cancer (prostate)	1.0 (1)	0.0 (0)	
Other	8.0 (8)	1.3 (1)	
Number of medications			
Mean ± SD	0.8 ± 1.0	0.4 ± 0.8	˂ 0.01
Tobacco use % (n)			
Smoking	6.0 (6)	2.6 (2)	NS
Use of snuff	11.0 (11)	7.9 (6)	NS
Time since COVID-19 diagnosis (months)			
Mean ± SD	12.4 ± 7.2	–	–
Range	(2.0–39.0)		
Severity of COVID-19 disease			
Mild	46.0 (46)		
Moderate	52.0 (52)	–	–
Severe	1.0 (1)		

Mann–Whitney U test, Chi-square, Fisher's exact test, NS = Not significant.

### Assessment of VAS self-reported smell and taste perceptions

**Figure 1:**
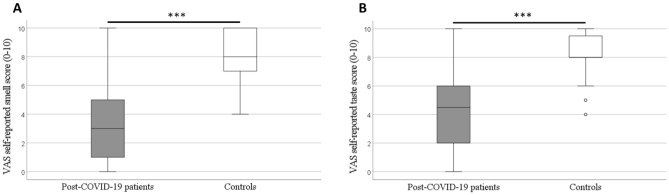
Boxplots illustrating (**A**) VAS self-reported smell score and (**B**) VAS self-reported taste score, in post-COVID-19 patients and controls. Mann–Whitney U test, ***p < 0.001. The circles in the figure represent outliers.

The onset of chemosensory loss was about 5 days after the COVID-19 infection in 90.0% of the patients, while the remaining 10.0% of patients could not remember the time of onset of their chemosensory loss. These patients reported complete and sudden chemosensory loss. Complete loss of smell was only reported in 9.0% of the patients, while 81.0% reported complete loss of smell and taste. The VAS self-reported smell score (median (IQR), range) was significantly lower in the patient group (3.0 (1.0–5.0), 0.0–10.0) than in the control group (8.0 (7.0–10.0), 4.0–10.0), Fig. 1A. The VAS self-reported taste score (median (IQR), range) was also significantly lower in the patient group (4.5 (2.0–6.0), 0.0–10.0) than in the control group (8.0 (8.0–9.7), 4.0–10.0), Fig. 1B. Median VAS self-reported smell and taste scores in the patient group were below the cut-off point (5.0) indicating low sense of smell and taste.

### Assessment of olfactory and gustatory functions

**Figure 2:**
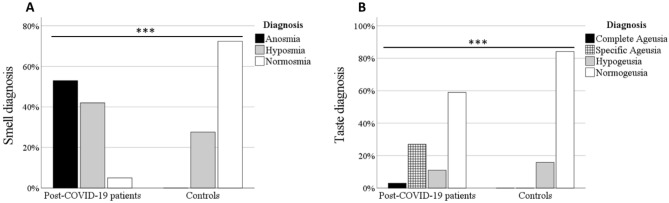
Histogram illustrating (**A**) smell diagnosis and (**B**) taste diagnosis, in post-COVID-19 patients and controls. Fisher’s exact test, ***p < 0.001.

The results of the objective measurements of smell and taste (Sniffin' Sticks test and Taste Strips test) are presented in Fig. 2. The prevalence of anosmia (53.0%) and hyposmia (42.0%) were significantly higher among post-COVID-19 patients than controls (anosmia (0.0%), hyposmia (27.6%)). The prevalence of ageusia (3.0%), specific ageusia (27.0%) and hypogeusia (11.0%) was also significantly higher among post-COVID-19 patients than controls (ageusia (0.0%), specific ageusia (0.0%), hypogeusia (15.8%)).

### Assessment of self-reported parosmia, dysgeusia, dysesthesia and xerostomia

**Figure 3:**
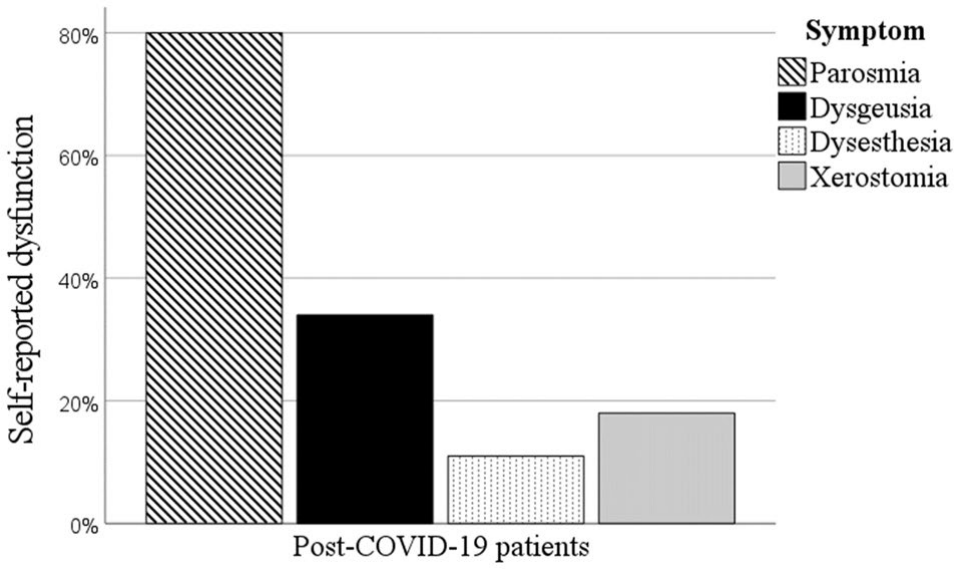
Histogram illustrating self-reported parosmia, dysgeusia, dysesthesia and xerostomia in post-COVID-19 patients.

Fisher's exact test showed a significantly higher prevalence of parosmia (80.0% vs 0.0%, *p* < 0.001), dysgeusia (34.0% vs 0.0%, *p* < 0.001), dysesthesia (11.0% vs 0.0%, *p* < 0.01) and xerostomia (dry mouth) (18.0% vs 0.0%, *p* < 0.001) among post-COVID-19 patients compared to controls who did not report any of these disturbances, Fig. 3.

The results from the follow-up questions regarding a) how often the dysfunctions were experienced and b) whether they were related to meals, are presented in Table 2**.** Among patients with dysgeusia, over 55.0% reported metallic taste, 20.0% reported bitter taste, and more than 17.0% reported rotten and harsh taste experience. “Other” tastes were reported in almost 9.0%, and salty in nearly 3.0% of the dysgeusic patients. There were some missing data in the follow-up questions regarding parosmia, dysgeusia, dysesthesia and xerostomia.

**Table 2:** Dysfunction characteristics of post-COVID-19 patients with self-reported parosmia, dysgeusia and dysesthesia.

Self-reported symptoms:	Parosmia (n = 80) % (n)	Dysgeusia (n = 34) % (n)	Dysesthesia (n = 11) % (n)
How often?			
Constantly	42.5 (34)	17.6 (6)	18.1 (2)
Daily	13.7 (11)	14.7 (5)	18.1 (2)
Sometimes	13.7 (11)	32.3 (11)	45.4 (5)
In contact with certain odorants	18.7 (15)	–	–
Periodically	3.7 (3)	8.8 (3)	0.0 (0)
Missing data	7.5 (6)	26.4 (9)	18.1 (2)
Related to meals?			
During meals	81.2 (65)	14.7 (5)	9.0 (1)
In between meals	3.7 (3)	32.3 (11)	27.2 (3)
Missing data	15.0 (12)	52.9 (18)	63.6 (7)
Description of dysgeusia			
Metallic		55.8 (19)	
Rotten		17.6 (6)	
Harsh	-	17.6 (6)	–
Salty		2.9 (1)	
Bitter		20.5 (7)	
Other		8.8 (3)	
Burning sensation			
Anterior tongue			45.4 (5)
Whole tongue			18.1 (2)
Lateral tongue			9.0 (1)
Palate	–	–	27.2 (3)
Throat			18.1(2)
Lips			18.1(2)
Buccal mucosa			9.0 (1)
Other			9.0 (1)

### Assessment of combinations of dysfunctions

**Figure 4:**
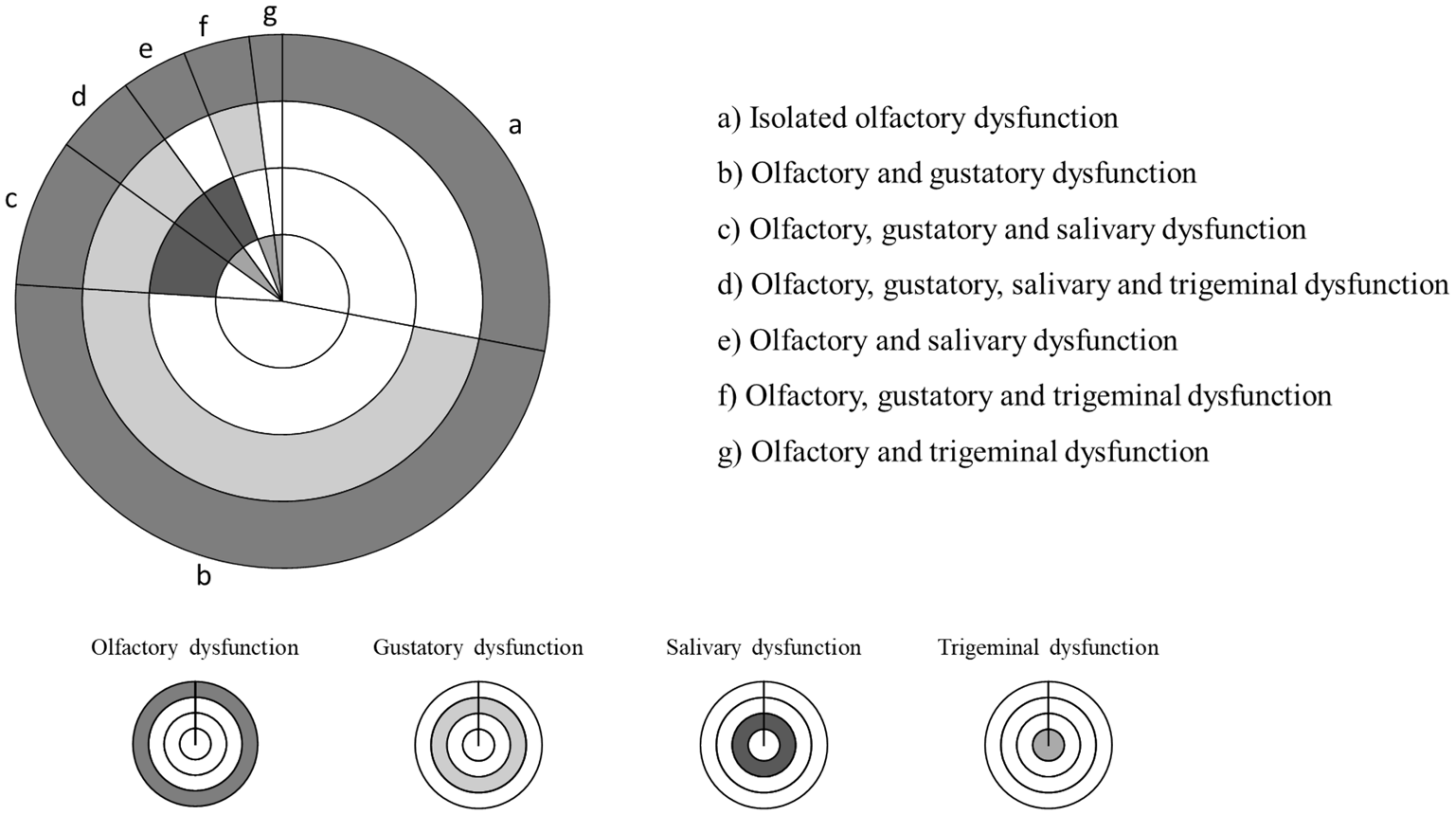
Pie chart showing distribution of patients with different combinations of dysfunctions (**a**–**g**).

In the post-COVID-19 patients, a combination of olfactory (smell) and gustatory (taste) dysfunction was the most prevalent complaint (48.0%), followed by isolated olfactory (smell) dysfunction (28.0%). Other combinations of dysfunctions found were: olfactory, gustatory and salivary dysfunction (9.0%), olfactory, gustatory, salivary and trigeminal dysfunction (5.0%), olfactory and salivary dysfunction (4.0%), olfactory, gustatory and trigeminal dysfunction (4.0%), and olfactory and trigeminal dysfunction (2.0%), Fig. 4.

### Assessment of specific taste ageusia

Among patients with specific ageusia, 66.7% had bitter taste ageusia, 37.0% had salt taste ageusia, 33.3% had sour taste ageusia and 3.7% had sweet taste ageusia. Moreover, specific ageusia was observed for one taste quality in 66.7%, in two taste qualities in 26.0% and in three taste qualities in 7.4% of the post-COVID-19 patients.

### Assessment of parosmia

The time of onset of parosmia was 4.6 ± 1.9 (mean ± SD) months after the COVID-19 infection. Patients reported that many food items, detergent products, hygiene articles and body odors had the same distorted smell. The smell was described as a very characteristic smell experienced as unpleasant, unfamiliar and indescribable. Some of the patients' descriptions of this smell were: “bonfire”, “chemical smell”, “sewage”, “smoke”, “burnt rubber”, “burnt plastic”, “rotten”, “fried liver”, “old sesame oil”, “unclean ice in the freezer”, “dead badger”, and “COVID-smell”. An overview of odors experienced as parosmic is shown in Table 3. Coffee was the most common distorted smell experienced by the patients in this study. More than 50.0% of patients experienced coffee as parosmic. In addition, many other types of drinks and beverages were experienced to some extent as parosmic, ranging from almost 30.0% to over 40.0%. More than 40.0% of the patients experienced bell peppers, garlic, onion, and cucumber as parosmic, and banana, was the most common parosmic fruit, reported by over 25.0% of the patients. The prevalence of distorted smell of egg, meat, poultry, and fish varied between 30.0 and 50.0%. Other food items like chocolate, nuts and bread were experienced parosmic in 20.0–30.0% of the patients. Hygiene articles like shampoo, conditioner, toothpaste, and detergents, were reported parosmic in more than 40.0% of the patients. Patients reported their own body odors such as sweat, urine and feces as parosmic in 32.0% to 45.0% of cases. Others body odors were perceived parosmic in over 20.0% of patients.

**Table 3:** Percentage of distorted odorants in post-COVID-19 patients with parosmia, specific ageusia for bitter taste, specific ageusia for salt taste and specific ageusia for sour taste.

Categories of odorants	Post-COVID-19 patients with parosmia (n = 80)	Post-COVID-19 patients with specific ageusia for bitter taste (n = 18)	Post-COVID-19 patients with specific ageusia for salt taste (n = 10)	Post-COVID-19 patients with specific ageusia for sour taste (n = 9)
	% (n)	% (n)	% (n)	% (n)
Drinks and beverages				
Coffee	56.4 (44)	33.3 (6)	50.0 (5)	33.3 (3)
Coke	42.3 (33)	22.2 (4)	40.0 (4)	22.2 (2)
Orange juice	39.7 (31)	16.7 (3)	20.0 (2)	33.3 (3)
Wine	37.2 (29)	11.1 (2)	20.0 (2)	22.2 (2)
Milk	30.8 (24)	11.1 (2)	20.0 (2)	11.1 (1)
Cold drink	29.5 (23)	5.6 (1)	10.0 (1)	33.3 (3)
Beer	28.3 (22)	5.6 (1)	10.0 (1)	11.1 (1)
Fruits and vegetables				
Bell pepper	46.2 (36)	33.3 (6)	20.0 (2)	33.3 (3)
Garlic	44.9 (35)	27.8 (5)	30.0 (3)	33.3 (3)
Onion	41.0 (32)	11.1 (2)	30.0 (3)	11.1 (1)
Cucumber	39.7 (31)	22.2 (4)	20.0 (2)	22.2 (2)
Celery	30.8 (24)	22.2 (4)	20.0 (2)	22.2 (2)
Tomato	28.2 (22)	11.1 (2)	10.0 (1)	11.1 (1)
Banana	25.6 (20)	11,1 (2)	0.0 (0)	0.0 (0)
Potato	21.8 (17)	11.1 (2)	10.0 (1)	0.0 (0)
Meat, fish and egg				
Egg	47.4 (37)	33.3 (6)	50.0 (5)	33.3 (3)
Meat	44.9 (35)	22.2 (4)	40.0 (4)	22.2 (2)
Chicken	37.2 (29)	22.2 (4)	20.0 (2)	11.1 (1)
Ham	34.6 (27)	16.7 (3)	30.0 (3)	0.0 (0)
Bacon	33.3 (26)	16.7 (3)	30.0 (3)	0.0 (0)
Fish	26.9 (21)	0.0 (0)	20.0 (2)	11.1 (1)
Other food items				
Chocolate	29.5 (23)	0.0 (0)	30.0 (3)	11.1 (1)
Nuts	28.2 (22)	11.1 (2)	10.0 (1)	22.2 (2)
Bread	23.1 (18)	0.0 (0)	10.0 (1)	11.1 (1)
Hygiene articles				
Shampoo/Conditioner	47.4 (37)	38.9 (7)	20.0 (2)	33.3 (3)
Toothpaste	44.9 (35)	16.7 (3)	20.0 (2)	22.2 (2)
Soap	28.2 (22)	38.9 (7)	40.0 (4)	33.3 (3)
Deodorant	28.2 (22)	33.3 (6)	20.0 (2)	33.3 (3)
Detergents				
Dishwasher soap	41.0 (32)	27.8 (5)	20.0 (2)	33.3 (3)
Detergent powder	38.5 (30)	27.8 (5)	20.0 (2)	22.2 (2)
Own body odors				
Feces	44.9 (35)	44.4 (8)	50.0 (5)	33.3 (3)
Urine	41.0 (32)	22.2 (4)	20.0 (2)	22.2 (2)
Sweat	32.1 (25)	5.6 (1)	20.0 (2)	11.1 (1)
Others body odor				
Partner	26.9 (21)	11.1 (2)	10.0 (1)	11.1 (1)
Children	23.1 (18)	16.7 (3)	0.0 (0)	0.0 (0)

### Possible associations between the chemosensory, trigeminal, and salivary dysfunctions

**Figure 5:**
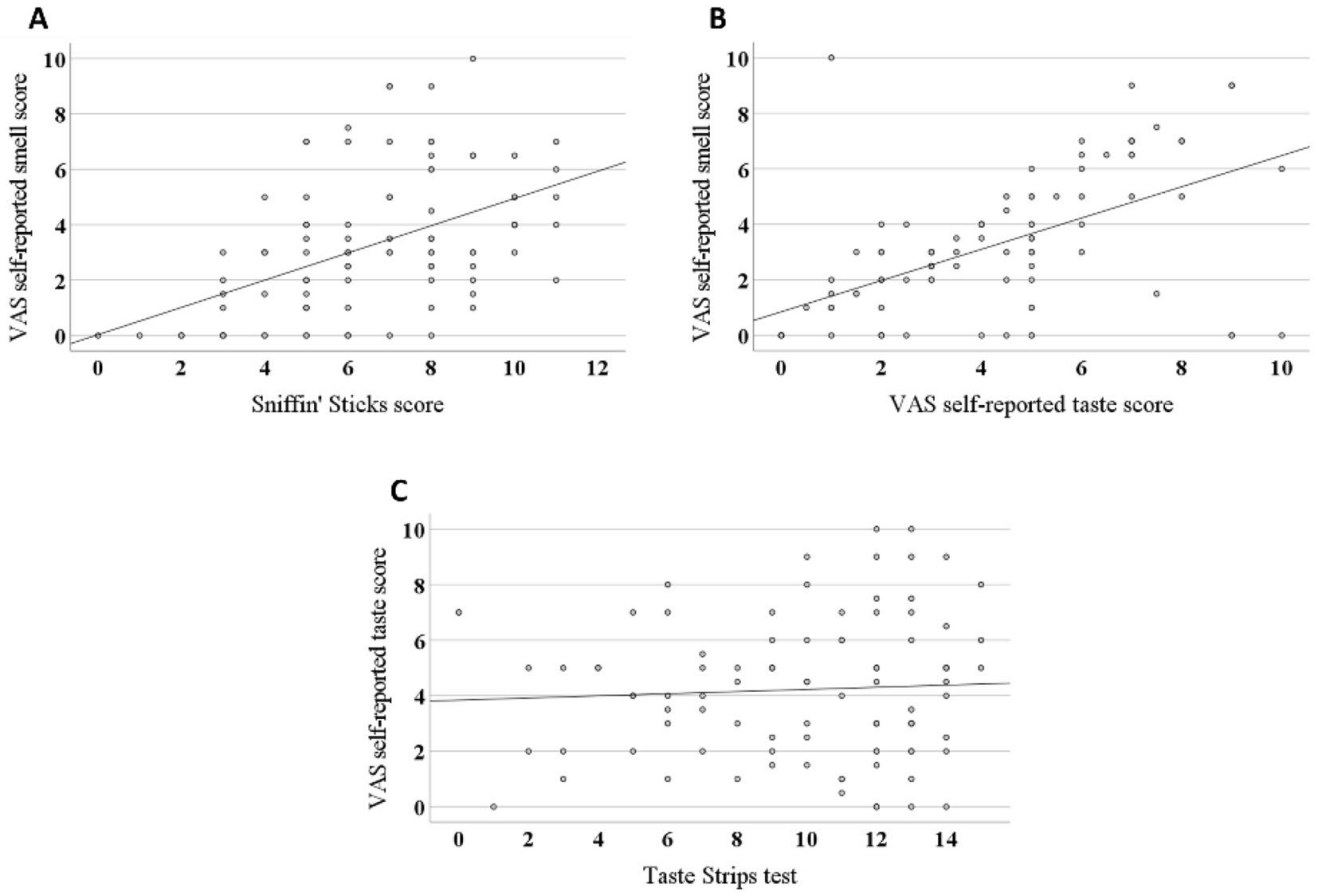
Scatter plots illustrating correlations between (**A**) VAS self-reported smell score and Sniffin' Sticks score (r = 0.5), (**B**) VAS self-reported smell score and VAS self-reported taste score (r = 0.6), and (**C**) VAS self-reported taste score and Taste Strips score (r = 0.06), Pearson's correlation coefficient test.

There were significant correlations between (1) VAS self-reported smell score and the measured Sniffin' Sticks score, Fig. 5A and (2) VAS self-reported smell score and VAS self-reported taste score Fig. 5B, but not between VAS self-reported taste score and the measured Taste Strips score, Fig. 5C. These results suggest that the olfactory dysfunction was recognized not only as a smell dysfunction, but also as a taste dysfunction by many patients.

Chi-square test of independence showed a significant positive dependence between dysgeusia and xerostomia (χ^2^ = 4.5, *p* ˂ 0.05). No significant correlations were found between trigeminal dysfunction and the other dysfunctions.

## Discussion

The major findings in the present study were that (1) post-COVID-19 patients suffered from various combinations of olfactory, gustatory, salivary, and trigeminal dysfunctions, (2) all taste qualities were compromised including specific ageusia for certain taste qualities, and (iii) most of the odors in our normal daily environment were found to be severely distorted.

In this study, seven different combinations of dysfunctions were found, with olfactory and gustatory dysfunction being the most prevalent, followed by isolated olfactory dysfunction. These findings are consistent with other studies that have reported olfactory, gustatory and trigeminal dysfunctions among COVID-19 patients^34,36,37,51,52^. Findings from this study showed that nearly one-fifth of post-COVID-19 patients complained of xerostomia which is consistent with other studies^45,53^. The ACE2 positive cells are known to be sensitive to SARS-CoV-2 virus infection, and it has been suggested that low expression of ACE2 due to infection is associated with increased inflammation and disease^18^. It has also previously been shown that the ACE2-expressing epithelial cells within the salivary gland ducts are among the first to be targeted by the SARS-CoV virus^54^. It is therefore plausible to hypothesize that this is similar for the closely-related SARS-CoV-2 virus, and therefore could be a possible explanation for salivary dysfunction in post-COVID-19 patients. Adequate salivary secretion is important for chewing, swallowing, nutritional intake, and the maintenance of a healthy oral environment^27,55,56^. It is therefore important to examine salivary function in patients recovering from COVID-19.

The patients in this study reported sudden smell and taste loss, shortly after COVID-19 infection. No considerable association was found between the patients' VAS self-reported taste function and their measured taste function. Many patients believed that their sense of taste was compromised, while it was in fact their sense of smell that was compromised. This underscores the challenge of distinguishing between the sense of smell and the sense of taste, and the importance of examining the two senses separately using psychophysical tests. The sense of taste is more protected than the sense of smell, partly because while the taste cells are innervated by three different cranial nerves, the olfactory cells/bulb are innervated by only one cranial nerve, and are therefore more vulnerable. As a result, taste dysfunction is not seen as commonly as smell dysfunction. However, in the post-COVID-19 patients in this study, both quantitative and qualitative taste dysfunctions were observed. Ageusia and specific ageusia, which are rare conditions, were found in almost one-third of the patients. In this study, specific ageusia for bitter, salt, sour and sweet taste was found, consistent with other studies^39,40^. Findings from the present study revealed that about one-fifth of post-COVID-19 patients had no bitter taste sensation, about one-tenth had no salt or sour taste sensation, and only one patient reported no sensation for sweet taste. While in the present study, bitter taste ageusia was most common, other studies have reported specific ageusia for sour taste as the most prevalent^39,40^. Loss of perception of bitter taste may have serious implications as bitter taste warns about ingestion of potentially harmful, toxic, or poisonous chemical substances^57^. Perception of salt and sour taste helps maintain water and electrolyte equilibrium in humans. Inability to detect salt and sour taste may lead to overconsumption of these tastants subsequently leading to health problems. These findings suggest that taste receptor cells for bitter, salt, and sour taste were compromised among this patient group. According to current literature, bitter, sweet and umami taste are mediated through Type II taste cells^58^ and sour taste is mediated through Type III taste cells^59^. Interestingly, in the present study, sweet taste was not as impaired as bitter taste, although both sweet and bitter taste receptors are found in the same subtype of taste receptor cells. This suggests that sweet receptors may have in some way been protected compared to bitter taste receptors in this post-COVID-19 cohort. In the future, more molecular biological studies are needed to fully understand how SARS-CoV-2 affects the different taste receptor cells.

Parosmia and dysgeusia were common findings in this study. Four out of five patients complained of parosmia, in line with other studies^34,60^. These reports are consistent with the theory that parosmia is believed to be a part of the recovery process in patients with impaired olfactory function^61,62^. All patients reported the distorted smell and taste to be of an unappealing character. The smells of many food items, like meat, egg, fruits and vegetables, dairy products, spices, and beverages, were severely distorted, and were more pronounced during meals among most of the post-COVID-19 patients. More than two-fifths of the patients reported coffee, eggs, bell pepper, garlic, meat, coke, and onion to be the most frequently distorted food/drink. Parosmia-affected food items in post-COVID-19 patients have also been previously reported, with coffee, meat and onion being the most frequently distorted food in a list of 14 food items^35^. Although the list of items in that study varied somewhat from the present study, there are consistencies between the two studies, and all the food items were found to be distorted to some degree in both studies. Dysgeusia was found in one-third of the patients, in agreement with a previous study^44^. While the occurrence of parosmia in COVID-19 patients has been largely explored, little is published about the different types of dysgeusia that also exists in this patient group. Findings from this study showed that patients most frequently described their dysgeusia as metallic, followed by bitter, rotten, harsh, other, and salty. Another study also reported metallic taste as the most common characteristic of COVID-19 related dysgeusia, followed by the taste of soap^63^. Present study demonstrates that dysgeusia and dysesthesia are quite prevalent conditions in post-COVID-19 patients more than a year after the initial viral infection.

This study is unique in the fact that we have explored parosmia, dysgeusia and dysesthesia related to meals among post-COVID-19 patients. While parosmia was more pronounced during meals, dysgeusia and dysesthesia were more pronounced “in between meals”, suggesting that patients could be suffering throughout the day. Parosmia has been suggested to affect the patients' nutritional intake^36^. However, a combination of distorted smell and taste, burning sensation in the oral cavity and oral dryness may cause a lot more despair in the patients and affect their nutritional status, emotional health, and quality of life. Therefore, special attention should be given to simultaneously examine all the different functions of the oral cavity rather than exploring them separately. A multidisciplinary mindset is crucial for appropriate diagnostics and for providing patients with relevant treatment regimes.

Trigeminal dysfunction and xerostomia were always found in combination with smell and/or taste dysfunction in this group. These patients were primarily referred to the ICD because of altered smell and taste function, so the prevalence of dysesthesia and xerostomia related to post-COVID-19 may well be even higher. Public awareness regarding dysesthesia and xerostomia related to post-COVID-19 should be increased to assure adequate treatment for this patient group.

One of the limitations of this study was that the controls were not specifically recruited for this project. Given the notable prevalence of asymptomatic COVID-19 cases^3^, it was considered necessary to use healthy controls from a period preceding the COVID-19 era, ensuring their non-exposure to the SARS-CoV-2 virus and its' potential imprints. Another limitation was that quantitative olfactory function was measured using the Sniffin' Sticks, 12-pen identification test and not the Threshold, Discrimination, and Identification (TDI) test of Sniffin' Sticks that would have given more precise evaluations. Unfortunately, the TDI test was not possible due to time limitations. Some patients regarded their sense of smell to be weak, even though they managed to identify most of the odors in the objective identification test, suggesting a poor Threshold score and high Identification score. Sole use of the identification test for olfactory function measurement does not take into account the patient's threshold value and their discrimination ability. Furthermore, it is worth noting that some of the odors present in the 16-pen identification test of Sniffin' Sticks were unfamiliar for some of our patients (e.g., turpentine, sauerkraut), and could have produced false negative outcomes. Similar findings have also been reported in the Danish population^64^. Notably, the 12-pen test does not include these less familiar odors such as turpentine and sauerkraut. The third limitation was that phantosmia and phantogeusia were not evaluated in this study. It is essential to acknowledge the potential presence of phantosmia in patients experiencing parosmia and phantogeusia in patients reporting dysgeusia. Lastly, it was a limitation that the salivary secretion rate of participants was not measured in this study because of limited resources. It would have been valuable to explore whether SARS-CoV-2 virus affected the flow rate of stimulated and/or unstimulated whole saliva, and not just rely on the participants self-reporting of dry mouth. A previous study found hyposalivation related to the stimulated flow rate evaluation in post-COVID-19 patients, but the sample size was small^45^. More studies are, therefore, needed to fully understand the extent of salivary dysfunction in post-COVID-19 patients.

In conclusion, the present study showed that the post-COVID-19 patient cohort suffered from many different combinations of chemosensory and oral dysfunctions. Not only smell, but also taste was found to be distorted, which may affect the patients' oral health-related quality of life. Oral dryness and burning sensation in the oral cavity were also common findings. More research is needed to explore the extent of these dysfunctions in post-COVID-19 patients.

### Supplementary Information

Supplementary Information.

## Data Availability

Request for materials should be addressed to the corresponding author, P.B.S.
