# Publisher Correction: Post-COVID-19 patients suffer from chemosensory, trigeminal, and salivary dysfunctions

**DOI:** 10.1038/s41598-024-59915-6

**Published:** 2024-04-22

**Authors:** Åsmund Rogn, Janicke Liaaen Jensen, Per Ole Iversen, Preet Bano Singh

**Affiliations:** 1 Department of Cariology and Gerodontology, Faculty of Dentistry, University of Oslo, Geitmyrsveien 71, 0455 Oslo, Norway; 2 Department of Oral Surgery and Oral Medicine, Faculty of Dentistry, University of Oslo, Oslo, Norway; 3 Department of Nutrition, Faculty of Medicine, University of Oslo, Oslo, Norway; 4 Department of Haematology, Oslo University Hospital, Oslo, Norway

Correction to: *Scientific Reports* 10.1038/s41598-024-53919-y, published online 11 February 2024

The original version of this Article contained errors in the Methods section where the hyperlinks to the sections of the Oslo COVID-19 questionnaire were incorrect.

Under the subheading ‘The Oslo COVID-19 questionnaire’,

“In section “Introduction”, patients’ age, gender, occupational status, and use of tobacco was recorded. In section “Methods”, information about COVID-19 infection was obtained; date of diagnosis, mode of confirmation of diagnosis (PCR test, home test, antibody test or clinical symptoms), and course of illness (mild, moderate, or severe). Finally, time for onset of loss of smell and taste, burning sensation and oral dryness was recorded (number of days before or after the confirmation of COVID-19 infection). Possible aetiology of chemosensory, trigeminal and salivary dysfunctions was also recorded (other viral or bacterial infections, menopause, trauma in head and neck region, head and neck surgery, dental surgery). In section “Results”, more specific questions about parosmia, dysgeusia, dysesthesia, and oral dryness were recorded as described below”.

now reads:

“In section 1 of the questionnaire, patients’ age, gender, occupational status, and use of tobacco was recorded. In section 2, information about COVID-19 infection was obtained; date of diagnosis, mode of confirmation of diagnosis (PCR test, home test, antibody test or clinical symptoms), and course of illness (mild, moderate, or severe). Finally, time for onset of loss of smell and taste, burning sensation and oral dryness was recorded (number of days before or after the confirmation of COVID-19 infection). Possible aetiology of chemosensory, trigeminal and salivary dysfunctions was also recorded (other viral or bacterial infections, menopause, trauma in head and neck region, head and neck surgery, dental surgery). In section 3, more specific questions about parosmia, dysgeusia, dysesthesia, and oral dryness were recorded as described below”.

The original Article has been corrected.

