# Supplementary material for: Post-COVID-19 patients suffer from chemosensory, trigeminal, and salivary dysfunctions
Source: Sci Rep. 2024 Feb 11;14:3455. doi: 10.1038/s41598-024-53919-y (PMC10859368; doi:10.1038/s41598-024-53919-y)
Supplement: Supplementary file 1 — Supplementary Information. [file 41598_2024_53919_MOESM1_ESM.pdf]

Post-COVID-19 patients suffer from chemosensory, trigeminal and salivary dysfunctions

Åsmund Rogn[1], Janicke Liaaen Jensen[2], Per Ole Iversen[3,4], Preet Bano Singh[1]

[1]Department of Cariology and Gerodontology, Faculty of Dentistry, University of Oslo, Oslo, Norway

[2]Department of Oral Surgery and Oral Medicine, Faculty of Dentistry, University of Oslo, Oslo, Norway

[3]Department of Nutrition, Faculty of Medicine, University of Oslo, Oslo, Norway

[4]Department of Haematology, Oslo University Hospital, Oslo, Norway

Oslo COVID-19 questionnarie

Section 1: General Information

Date:

ID:

Age:

Gender:	Male	Female	Other
Smoker:	Yes	No	
Snuff user:	Yes	No	

Chronic diseases:

Daily use of medications:

Allergies:

Occupational status:	Full time work	Part time work	Temporary laid off	On sick leave	Retired	Student

Section 2: Questions related to COVID-19

When were you diagnosed with COVID-19?

Which test was used for diagnosis?	PCR test	Antibody test	Home test	Clinical symptoms only
Course of illness:		Mild	Moderate	Severe

Symptoms:

Day 1:

Day 2:

Day 3:

Day 4:

Day 5:

After two weeks:

After one month:

Other:

When did your sense of smell and taste return?

After two weeks:

After one month:

Other:

Status today:

Other aetiology

Did you experience trauma in head-neck region before the onset of symptoms? Yes No

If yes, describe:		
Did you undergo dental surgery before the onset of symptoms?	Yes	No
If yes, describe:		
Did you undergo surgery in head-neck region before the onset of symptoms?	Yes	No
If yes, describe:		
Were you undergoing hormonal changes at the time of onset of symptoms?	Yes	No
If yes, describe:		
Did you have other bacterial/viral infections before the onset of symptoms?	Yes	No
If yes, describe:		

Section 3

Dysgeusia

Do you experience bad taste on the tongue?			Yes			No
What kind of taste do you experience?	Metallic	Bitter	Rotten	Harsh	Other	
How often do you experience bad taste?	Constantly	Daily	Sometimes	Periodically	Other	
When is the bad taste most pronounced?		During meals	In between meals	Other		
Do you have to refrain from certain food items due to bad taste?			Yes			No
If yes, what kind of food items do you have to avoid?	Spicy	Sweet	Sour	Salty	Bitter	Other

Dysesthesia

Do you experience burning sensation in the mouth?			Yes			No
Where in your mouth do you experience burning?	Anterior tongue	Whole tongue	Lips	Palate	Other	

How often do you experience burning?	Constantly	Daily	Sometimes	Periodically	Other	
When is the burning most pronounced?		During meals	In between meals	Other		
Do you have to refrain from certain food items due to burning?			Yes			No
If yes, what kind of food items do you have to avoid?	Spicy	Sweet	Sour	Salty	Bitter	Other

Dry Mouth

Do you experience dry mouth?			Yes			No
If yes, is the dry mouth more pronounced after COVID-19?			Yes			No

Parosmia

Do you experience that the odors around you are distorted?			Yes			No
If yes, describe:						
How often do you experience that the odors are distorted?	Constantly	Daily	Sometimes	In contact with certain odorants	Other	
When is the distortion most pronounced?		During meals	In between meals	Other		

Choose one alternative

	No smell/taste	Distorted smell/taste	Reduced smell/taste	Normal smell/taste
**Drinks and beverages**				
Coffee				
Black soda (Coke, Pepsi)				

Orange juice
Wine
Milk
Soda (Fanta, Sprite)
Beer
**Fruits and vegetables**
Paprika
Garlic
Onion
Cucumber
Celery
Tomatoes
Bananas
Potatoes
**Meat, fish and egg**
Egg
Meat
Chicken
Ham
Bacon
Fish
**Other food items**
Chocolate
Nuts
Bread
**Hygiene articles**
Shampoo/Conditioner
Toothpaste
Soap
Deodorant
**Detergent**
Washing up liquid
Detergent powder
**Own body odor**
Feces

Urine
Sweat
**Others body odor**
Partner
Children
